# Response of cassava cultivars to African cassava mosaic virus infection across a range of inoculum doses and plant ages

**DOI:** 10.1371/journal.pone.0226783

**Published:** 2019-12-23

**Authors:** Jerome Anani Houngue, Justin S. Pita, Hermine Bille Ngalle, Martine Zandjanakou-Tachin, Apollin Fotso Kuate, Gilles Habib Todjro Cacaï, Joseph Martin Bell, Corneille Ahanhanzo

**Affiliations:** 1 Central Laboratory of Plant Biotechnology and Plant Breeding, Department of Genetics and Biotechnology, Faculty of Sciences and Techniques, University of Abomey-Calavi, Abomey-Calavi, Benin; 2 Laboratory of Genetics and Plant Breeding, Department of Plant Biology, Faculty of Science, University of Yaoundé 1, Yaoundé, Cameroon; 3 Laboratory of Plant Physiology, Université Felix Houphouët-Boigny, Abidjan, Cote d’Ivoire; 4 Laboratory of Molecular Plant Pathology, School of Horticulture and Green Space Management, National University of Agriculture, Porto-Novo, Benin; 5 International Institute of Tropical Agriculture (IITA), Nkolbisson, Yaoundé, Cameroon; Washington State University, UNITED STATES

## Abstract

Cassava production in Africa is constrained by cassava mosaic disease (CMD) that is caused by the *Cassava mosaic virus* (CMV). The aim of this study was to evaluate the responses of a range of commonly cultivated West African cassava cultivars to varying inoculum doses of *African cassava mosaic virus* (ACMV). We grafted 10 cultivars of cassava plants with different inoculum doses of CMV (namely two, four, or six CMD-infected buds) when the experimental plants were 8, 10, or 12 weeks old, using non-inoculated plants as controls. Three cultivars showed disease symptoms when grafted with two buds, and four cultivars showed disease symptoms when grafted with four or six buds. Most cultivars became symptomatic six weeks after inoculation, but one (‘TMS92/0326’) was symptomatic two weeks after inoculation, and two (‘Ntollo’ and ‘Excel’) were symptomatic after four weeks. Root weight tended to be lower in the six-bud than in the two-bud dose, and disease severity varied with plant age at inoculation. These results indicate that the level of CMD resistance in cassava cultivars varies with inoculum dose and timing of infection. This will allow appropriate cultivars to be deployed in each production zone of Africa in accordance with the prevalence of CMD.

## Introduction

Cassava (*Manihot esculenta* Crantz) is an important, carbohydrate-rich root crop cultivated throughout the tropics. This crop is prone to cassava mosaic disease (CMD) which is caused by several geminiviruses, making CMD the most important viral disease affecting cassava production in Africa [1]. Cassava mosaic geminivirus (CMG) infection of cassava plants reduces yields and, in highly susceptible cultivars, causes root losses of up to 100% [2]. These CMGs are principally transmitted through infected plant material, such as cuttings, and by the whitefly vector *Bemisia tabaci*, for which chemical control methods are inefficient and costly, and may cause other types of damage to the target plant. The resistant varieties adoption constitute the most effective solution to control the negative effects of CMD in cassava production [3, 4]. Several studies have evaluated the resistance of cassava cultivars to CMD [5, 6]. However, the infestation pressure is often underestimated or not taken into account because only whitefly transmits the disease during the evaluation period of typical field experiments [7]. Studies have also shown that the rate of CMD transmission by whitefly is relatively low and varies according to the whitefly population at an experimental site [8].

Furthermore, a plant infected at an older age may exhibit milder symptoms than one infected at a young age [9]. Thus, some cultivars with partial resistance to CMD reacted as susceptible under greenhouse inoculation, whereas in field infection they showed a “resistance” level comparable to other more highly resistant cultivars [10].

Although responses of cassava cultivars to natural levels of CMG infection have been evaluated in the field [11, 6], the impact on plant health tends to be underestimated in field studies, due to spatio-temporal variation in whitefly activity and variation in plant age at the time of infection. In field evaluation, intensity of inoculation, level of viral inoculum, and plant age at infestation are all unknown. In contrast, glasshouse studies allow the determination of inoculum dose-response effects on levels of resistance in cassava cultivars [6, 12] that have been shown to positively affect the likelihood of infection [13]. The age at which plants are most susceptible to CMGs and the optimum inoculum dose for the quantification of resistance to CMD remain unclear, but their elucidation could inform more effective cassava crop management. The synchronization between the inoculum dose, plant age at infestation and root number and weight in cassava is unknown, and the elucidation could help to establish good inoculation protocols for suitable resistance evaluation of cassava cultivars to CMD. For instance, under glasshouse, plant age at inoculation and inoculum dose per infected plant can all be manipulated by the researcher conducting the inoculation. Thus, the aims of this study were firstly, to estimate levels of resistance to CMD in a range of West African cassava cultivars, secondly to determine the influence of plant age on CMD susceptibility among cultivars, and thirdly to evaluate the effects of levels of infection on root development.

## Materials and methods

### Cassava plant material and inoculum source

We selected 10 cultivars of cassava of various origins, comprising five locally cultivated by famers (three from Benin and two from Cameroon), one landrace from Benin, and improved cultivars–two from Benin and two from Cameroon (Table 1). Cuttings of ‘Agric-rouge’, ‘Adjatidaho’, ‘Atinwewe’, ‘BEN86052’, ‘92B/0057’, and ‘TME7’ were obtained from tissue cultures of asymptomatic plants stored at Central Laboratory of Biotechnology and Plant Breeding of the University of Abomey-Calavi in Benin. The cuttings were certified by ‘Plant Protection Organization of Benin’ on N° 0002975/16/SPVCP/CP/AE-B (S1 File) before sent to the University of Yaoundé I. Further cuttings of asymptomatic ‘TMS92/0326’, ‘Excel’, ‘Oboul-doux’, and ‘Ntollo’ were taken *in situ* from field-grown plants of International Institute of Tropical Agriculture of Cameroon (IITA). Specific permission is not required for sample collection since the experiment constitute a collaborative research between the author and co-authors. The 15-cm-long cuttings were treated with hot water, as described by Zinga et al. [14], prior to planting as single stems in 4-L pots filled with sterile soil/manure mixture (1:1 v/v). The pots were irrigated to field capacity once per day until sprouting, and twice per week thereafter; cuttings were grown in a glasshouse maintained at 28 °C, with relative humidity >50%, and natural lighting with an approximate light/dark cycle of 12/12 h at the University of Yaoundé 1 in Cameroon.

**Table 1:** Characteristics of cassava cultivar planting material.

Cultivar	Origin	Type	Type of resistance genes
Agric-rouge	Benin	Local	CMD2
Adjatidaho	Benin	Local	Unknown
Atinwewe	Benin	Local	Unknown
BEN86052	Benin	Improved	Unknown
92B/0057	Benin	Improved	CMD2
Oboul-doux	Cameroon	Local	Unknown
TMS92/0326	Cameroon	Improved	Unknown
Excel	Cameroon	Improved	Unknown
Ntollo	Cameroon	Local	Unknown
TME7	Benin	Landrace	CMD2

**Fig 1:**
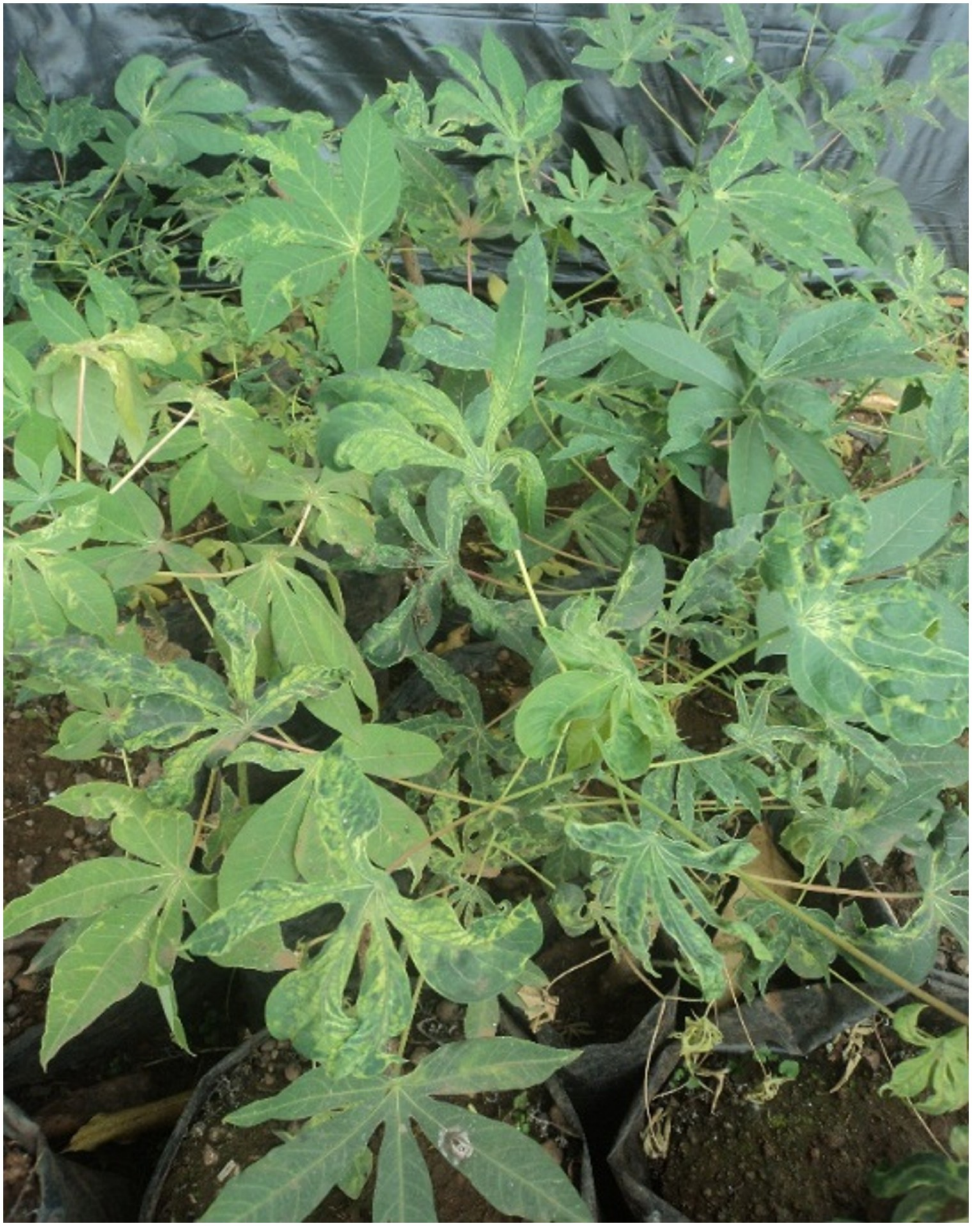
Cassava plants infected with cassava mosaic geminiviruses used as inoculum.

When assessing the presence of *African cassava mosaic virus* (ACMV) and *East African cassava mosaic virus* (EACMV) infection, plant material was sourced from cuttings at six weeks after planting and from cultivated plants of the highly CMD-susceptible Cameroon cultivar ‘Manioc de Table’ (Fig 1).

### Molecular analysis of CMD

The source plant for our work was the cultivar ‘Manioc de Table’. Fresh leaves were collected from the inoculated plants one month after grafting, and genomic DNA was extracted following the protocol described by Dellaporta et al. [15], with some modifications [16]. PCR analyses were performed using primers JSP1 and JSP2 (5′-ATGTCGAAGCGACCAGGAGAT-3′ and 5′-TGTTTATTAATTGCCAATACT-3′, respectively) to detect ACMV, and JSP1 and JSP3 (5′-ATGTCGAAGCGACCAGGAGAT-3′ and 5′-CCTTTATTAATTTGTCACTGC-3′, respectively) for detecting EACMV, following the protocol described by Pita et al. [17]. PCR products were visualized on 1.8% agarose gel using EZ-Vision (VWR International, Radnor, PA, USA) and visualized under UV light.

### CMD inoculation

Grafting technique has been successfully used for artificial inoculation of CMVs in cassava plants [8, 18], so we used side cleft grafting, in which a tangential cleft was made in the main stem, close to a leaf node, following the approach reported by Wagaba et al. [18]. Axillary buds (3–6 mm), with the petiole and leaf attached, were excised from virus-free plants to the sixth nodes from the apex. Axillary buds of a similar size (3–6 mm) were excised from inoculum source plants and inserted under the first five apical nodes of the virus-free plants to a depth of 2 mm, to expose the cambium layer, by making a triangular-shaped cut using a double-edged razor blade. The grafts were secured tightly, using Parafilm, to promote union and prevent desiccation. A maximum of six buds attached with petiole and leaf were removed from control plants to quantify photosynthetic effects of the virus on root storage.

**Fig 2:**
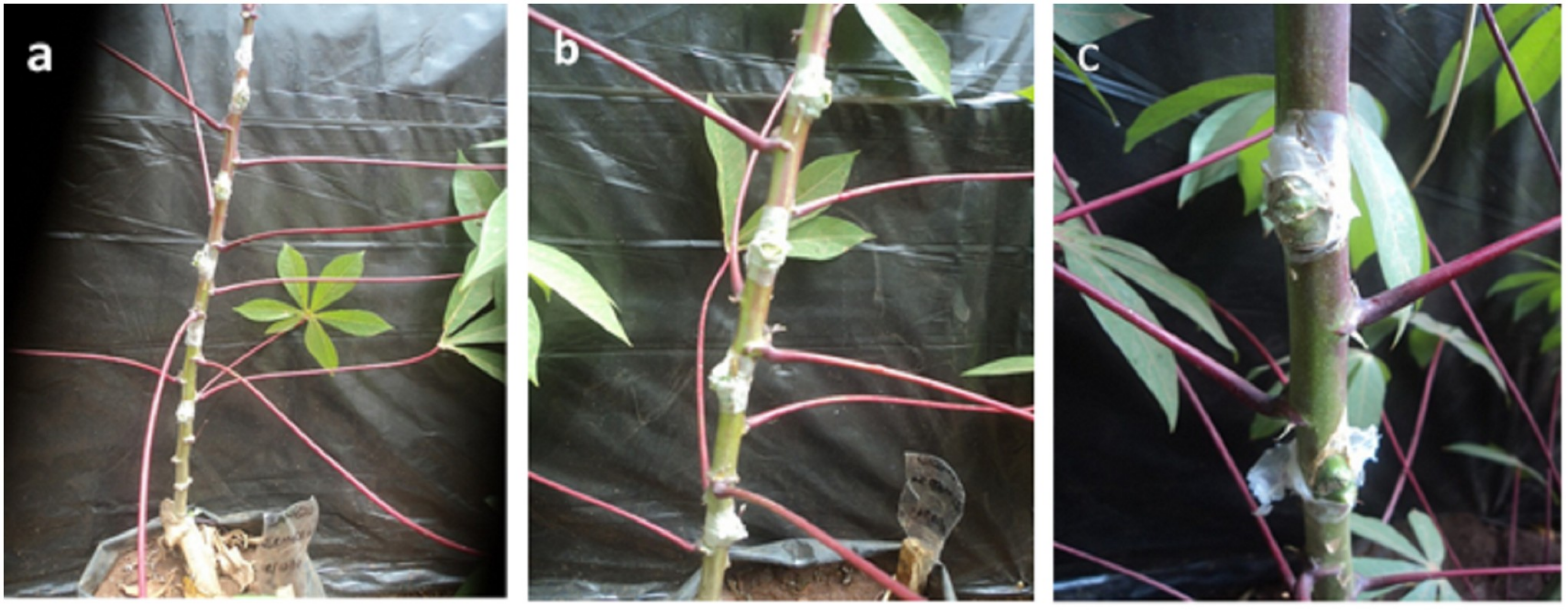
Cassava plants of BEN86052 inoculated by grafting. Six buds (**a**), four buds (**b**), and two buds (**c**).

Treatments comprised six replicates of two, four, or six infected buds (2B, 4B, and 6B, respectively) grafted to 8-week, 10-week, or 12-week-old (8W, 10W, and 12W, respectively) plants of each cultivar (Fig 2) that were arranged using a full-factorial design in a glasshouse (8W2B, 8W4B, 8W6B, 10W2B, 10W4B, 10W6B, 12W2B, 12W4B, and 12W6B), with two virus-free plants of each cultivar as non-inoculated controls. The experiment was repeated twice (October 2016 and April 2017).

### Disease severity

**Fig 3:**
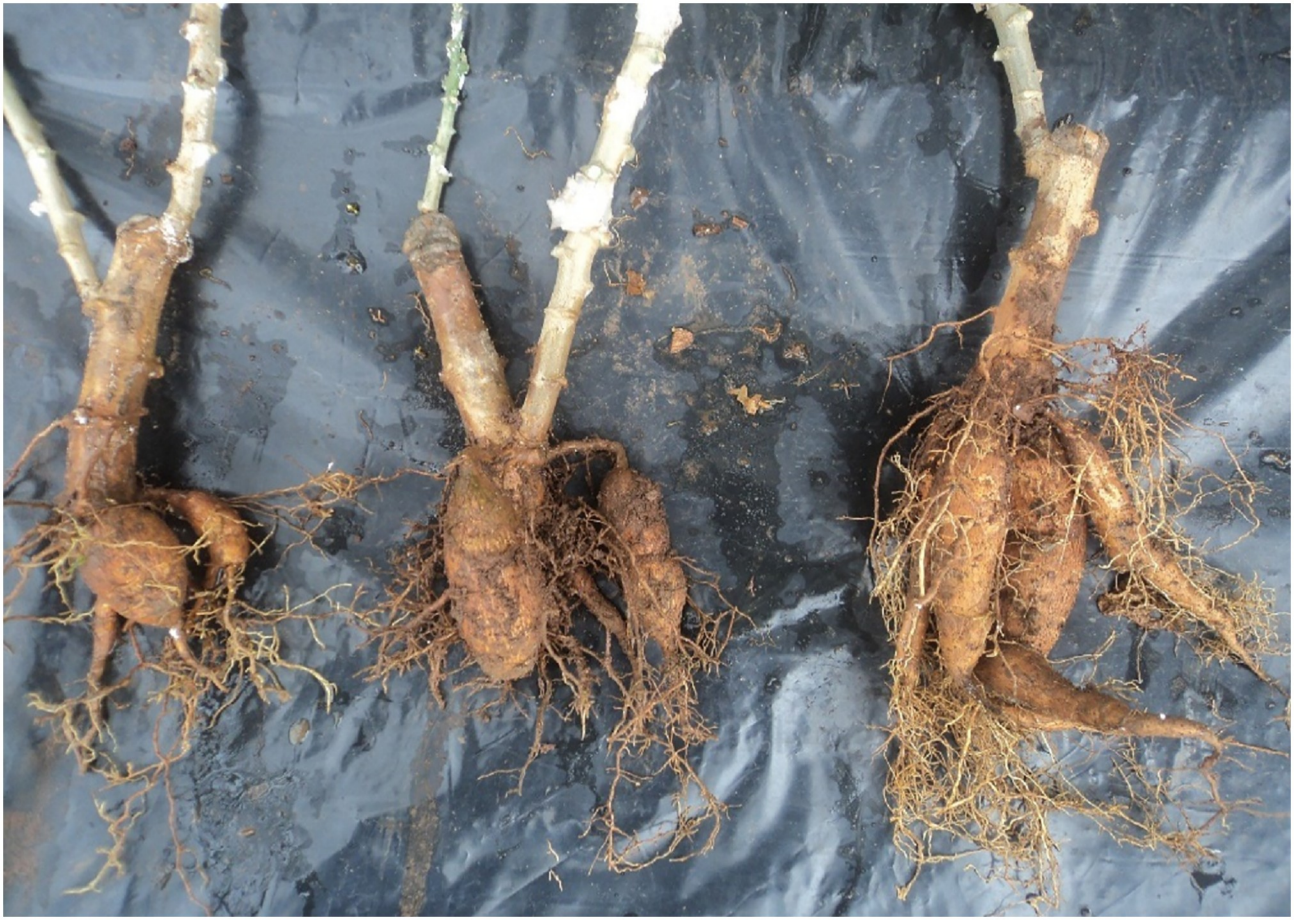
Cassava roots system, according to inoculum dose of inoculated plants of BEN86052, 3 months after inoculation.

Plants were visually assessed for CMD leaf symptom severity for 3 months, every 2 weeks after inoculation, using a scale of 1–5 described by Terry [19], where 1 = No leaves with symptoms characteristic of CMD; 2 = Slight curl characteristic of CMD seen on leaves; 3 = CMD curling easily observable on leaves; 4 = CMD curling seen on many leaves; 5 = Very severe curling and leaf wilt. Storage roots were removed at 24 weeks after planting (Fig 3), and root number and weight were recorded.

### Data analysis

Analyses were performed using XLSTAT v.2014 (Addinsoft, Paris, France). Infection level, age at inoculation and their influence on disease severity were used as metrics for disease susceptibility among the cultivars; these were tested using principal component analysis (PCA). The effect of plant age at inoculation and inoculation dose (number of buds) on disease severity and the number of storage roots among the cultivars were tested using analysis of variance, and inoculum dose-response effects on the non-normally distributed storage root weight were tested using gamma regression.

## Results

### Presence of CMD viruses in inoculum source and cassava cultivars after grafting

**Fig 4:**
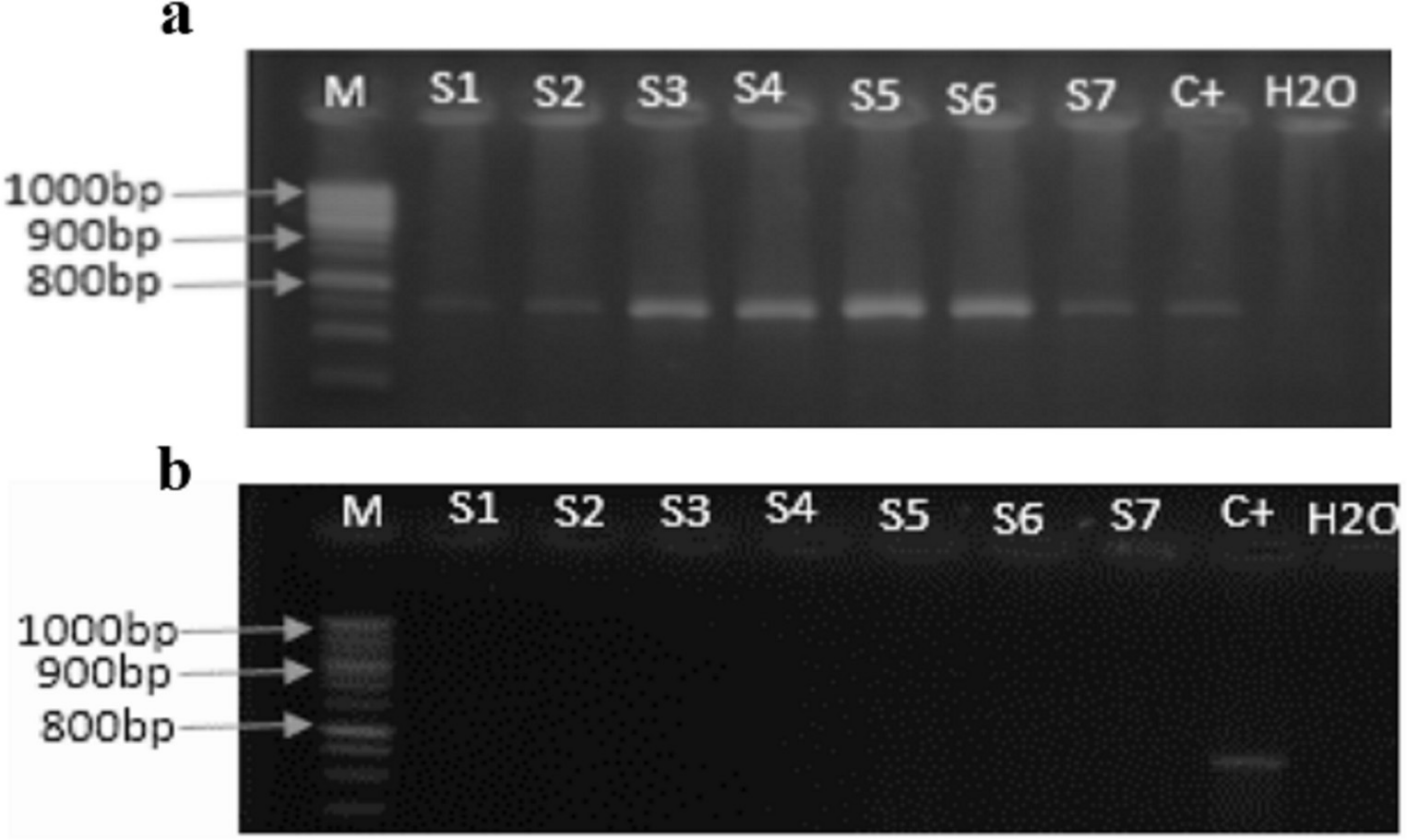
Electrophoresis gels showing CMD virus in inoculum source plants. Amplification of coat protein sequence of ACMV (**a**) and EACMV (**b**). M: ladder (1000 bp– 100 bp); S1 –S7: leaf sample tested; C^+^: positive control; H20: grade water used as negative control.

The PCR products showed the presence of ACMV in seven biological replicates (plants) of the source cultivar ‘Manioc de table’ (Fig 4a) but no plants tested positive for EACMV (Fig 4b). After the grafting, the plants of different cultivars responded differentially to the virus. Most of tested plants were positive to ACMV (S2 File.) according to the genotypes, the age and the inoculum dose (Table 2).

**Table 2:** Presence of ACMV in cultivars following inoculation.

Cultivars	Treatments								
	8W2B	8W4B	8W6B	10W2B	10W4B	10W6B	12W2B	12W4B	12W6B
Agric-rouge	−	−	−	−	−	−	−	−	+
Adjatidaho	−	−	+	−	+	+	−	+	+
Atinwewe	−	−	−	−	−	−	−	−	−
BEN86052	+	+	+	+	+	+	−	+	+
92B/0057	+	+	+	−	+	+	+	+	+
Oboul-doux	+	+	+	+	+	+	+	+	+
TMS92/0326	−	+	+	+	+	+	−	−	−
Excel	+	+	+	+	+	+	−	+	+
Ntollo	+	+	+	+	+	+	+	+	+
TME7	−	−	−	−	−	−	−	−	−

−: negative; +: positive; W: week; B: buds; ACMV: African cassava mosaic virus; 8W2B: Plants inoculated at the age of eight weeks with two buds; 8W4B: Plants inoculated at the age of eight weeks with four buds; 8W6B: Plants inoculated at the age of eight weeks with six buds; 10W2B: Plants inoculated at the age of ten weeks with two buds; 10W4B: Plants inoculated at the age of ten weeks with four buds; 10W6B: Plants inoculated at the age of ten weeks with six buds; 12W2B: Plants inoculated at the age of twelve weeks with two buds; 12W4B: Plants inoculated at the age of twelve weeks with four buds; 12W6B: Plants inoculated at the age of twelve weeks with six buds.

### Cassava cultivar susceptibility

The principal components PC1 and PC2 of the principal components analysis explained 77.86% of the variation in disease severity in the cultivars among treatments. Disease severity effects of treatments 8W4B, 8W6B, 10W2B, 10W4B, 10W6B, 12W4B, and 12W6B were associated with PC1, and those of treatments 8W2B and 12W2B were associated with PC2 (S1 Table).

**Fig 5:**
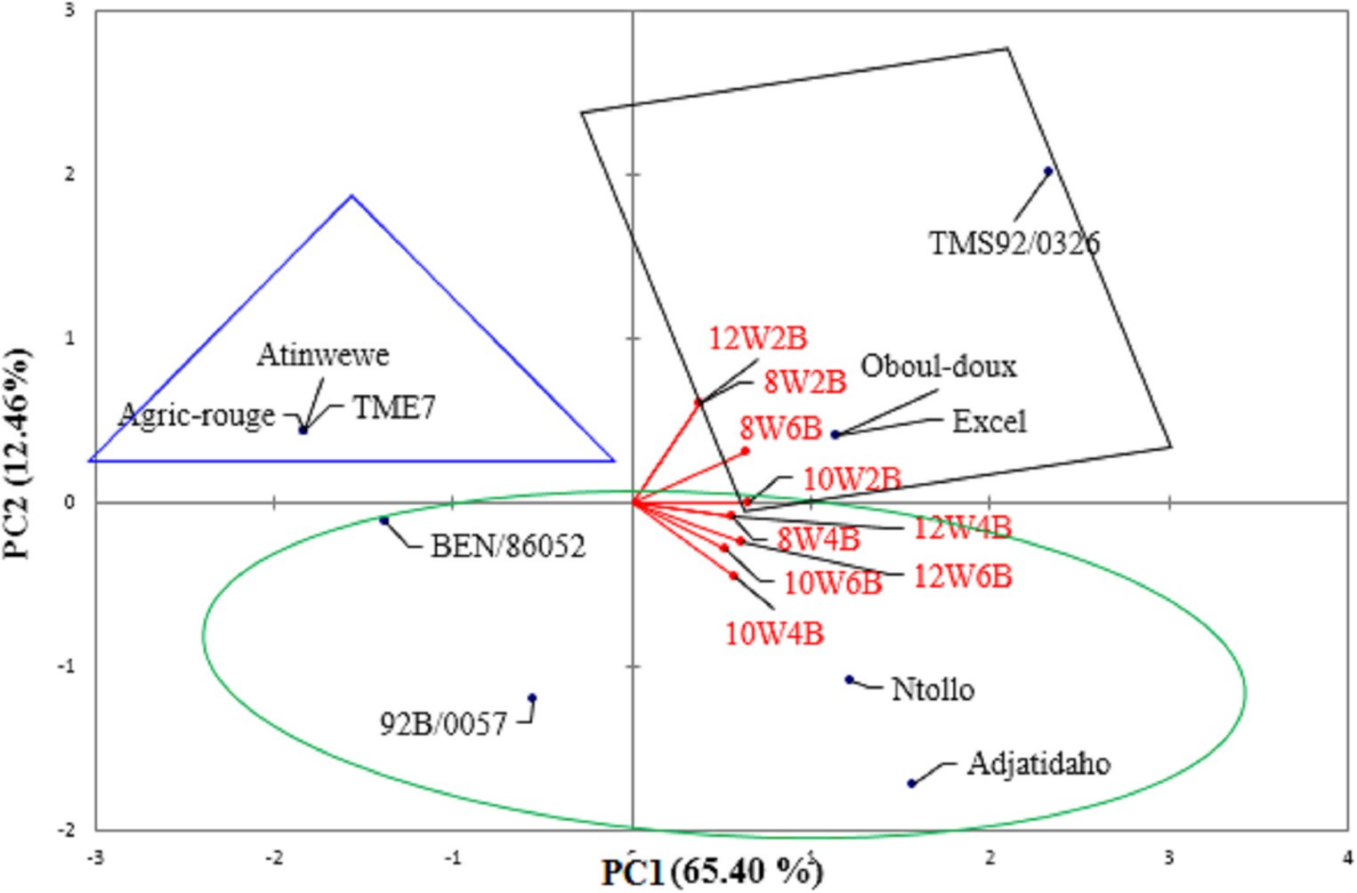
PCA biplot of disease severity in cassava cultivars along principal components PC1 and PC2.

Disease severity in three cultivars (‘TMS92/0326’, ‘Oboul-doux’, and ‘Excel’) was associated with treatments 8W2B, 8W6B, 10W2B, and 12W2B. In cultivars ‘Ntollo’, ‘Adjatidaho’, ‘92B/0057’, and ‘BEN/86052’, disease severity was associated with treatments 8W4B, 10W2B, 10W4B, 10W6B, 12W4B, and 12W6B. Disease severity in cultivars ‘Agric-rouge’, ‘TME7’, and ‘Atinwewe’ was not associated with any treatment (Fig 5). Based on these results, disease susceptibility of cultivars may be classified by plant age at inoculation (W: weeks) and inoculum dose (B: buds).

### Effect of plant age at inoculation and inoculum dose on disease severity

**Fig 6:**
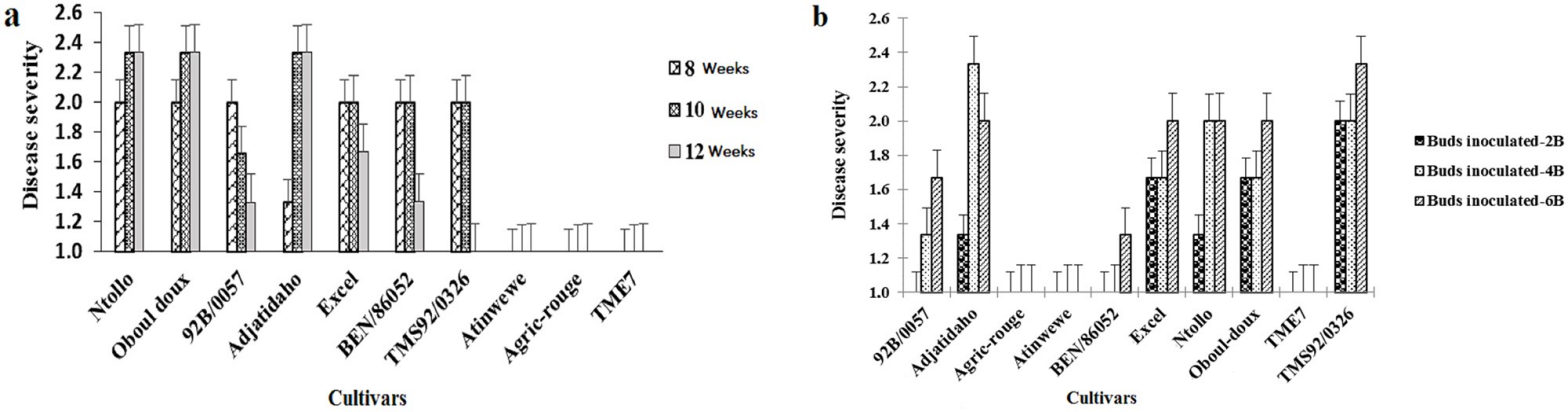
Variation in cassava mosaic disease severity among cassava cultivars: Effect of age at inoculation (a) and inoculum dose (b).

There were differences (*P* < 0.0001) in disease severity among cultivars at different ages of inoculation and inoculum dose (Table 3). There were no symptoms of disease in ‘Atinwewe’, ‘Agric-rouge’, or ‘TME7’, regardless of age at inoculation (Fig 6a) and inoculum dose (Fig 6b), whereas symptoms of disease were apparent in ‘Oboul-doux’, ‘Ntollo’, ‘92B/0057’, ‘TMS92/0326’, ‘Excel’, ‘BEN/86052’, and ‘Adjatidaho’, and varied with age at inoculation (Fig 6a) and inoculum dose (Fig 6b). Earlier age at inoculation did not always correspond to greater severity score: for example, disease symptoms in ‘Oboul-doux’, ‘Ntollo’, and ‘Adjatidaho’ were more severe in plants inoculated at 10 or 12 weeks than at 8 weeks of age (Fig 6a). We found that the reverse was true for ‘92B/0057’, ‘TMS92/0326’, ‘Excel’, and ‘BEN/86052’.

**Table 3:** Analysis of variance of CMD severity for age at inoculation and inoculum dose.

Source	DF	Sum of squares	Mean squares	*F*	*P*
Cultivars	9	15.1222	1.6802	13.7474	< 0.0001
Age at inoculation	2	1.0888	0.5444	4.4545	0.0157
Buds inoculated	2	1.6888	0.8444	6.9090	0.0019
Cultivars x age at inoculation	18	2.9111	0.1617	1.3232	0.2067
Cultivars x buds inoculated	18	2.3111	0.1283	1.0505	0.4216
Age at inoculation x buds inoculated	4	0.1777	0.0444	0.1531	0.9610

### Effect of inoculum dose on storage roots

Overall, there were differences in the number of storage roots among cultivars inoculated with two (*P* ˂ 0.0001), four (*P* = 0.0001), and six buds (*P* = 0.0007) (S2 Table), where numbers were lower in infected plants; effects were greater at higher doses (Table 4). Overall, the greatest loss in storage root number was in ‘Oboul-doux’ in the six-bud treatment, and the lowest loss was in ‘Adjatidaho’ in the two-bud treatment.

**Table 4:** Effect of viral dose (number of buds) on mean number of storage roots per plant.

	Number of storage roots						
	Six buds		Four buds		Two buds		Control
Cultivar	Mean ± SE	Loss %	Mean ± SE	Loss %	Mean ± SE	Loss %	Mean ± SE
Adjatidaho	3.33 ± 0.55	52.85	5.66 ± 0.84	19.14	6.33 ± 0.55	4.85	7.00 ± 0.36
BEN/86052	4.33 ± 0.84	50.00	4.66 ± 0.21	46.18	7.33 ± 0.76	15.35	8.66 ± 0.21
92B/0057	3.33 ± 0.21	65.52	5.00 ± 0.36	48.24	6.33 ± 0.83	34.55	9.66 ± 0.42
Atinwewe	2.33 ± 0.84	66.71	3.66 ± 0.76	47.71	4.33 ± 0.21	38.14	7.00 ± 0.84
Agric-rouge	4.00 ± 0.73	57.12	5.66 ± 1.11	39.33	6.00 ± 1.09	35.69	9.33 ± 0.76
TME7	3.66 ± 0.21	52.21	5.00 ± 0.25	34.72	5.00 ± 0.36	34.72	7.66 ± 0.21
Excel	2.66 ± 0.21	68.06	3.33 ± 0.21	60.02	2.66 ± 0.21	68.06	8.33 ± 0.84
Oboul-doux	1.66 ± 0.42	72.33	2.33 ± 0.55	61.16	1.66 ± 0.42	72.33	6.00 ± 1.09
TMS92/0326	2.00 ± 0.21	68.40	2.66 ± 0.21	57.97	2.33 ± 0.21	63.19	6.33 ± 0.42
Ntollo	1.33 ± 0.21	83.37	2.66 ± 0.55	66.75	3.00 ± 0.36	62.5	8.00 ± 1.31
**Overall**	**2.86**	**63.66**	**4.06**	**48.12**	**4.53**	**42.93**	**7.79**
**LSD**	**1.454**	**–**	**1.677**	**–**	**1.550**	**–**	**2.108**
***R***^**2**^	**0.650**	**–**	**0.46**	**–**	**0.70**	**–**	**0.497**
***F***	**3.97**	**–**	**4.72**	**–**	**13.28**	**–**	**3.03**
***P***	**0.0007**	**–**	**0.0001**	**–**	**˂0.0001**	**–**	**0.0057**

Cultivar ‘BEN/86052’ produced the greatest mean number of roots in the two-bud treatment, while ‘Oboul-doux’ had the lowest number of storage roots. There was no overall effect of the four-bud treatment on root number, in which ‘Adjatidaho’ had the greatest number of storage roots and ‘Oboul-doux’ had the lowest number. The greatest relative loss of number of storage roots was for ‘Ntollo’ and the lowest was for ‘Adjatidaho’. The greatest mean number of storage roots in the six-bud treatment was for ‘BEN/86052’ and the lowest number was for ‘Ntollo’.

There were differences in storage root weight among cultivars inoculated with two, four, and six buds (P < 0.05) (S3 Table), with storage root weight tending to be lower than in the non-inoculated controls (Table 5). In the six-bud treatment, the greatest storage root weight was for ‘Adjatidaho’ and lowest for ‘Excel’. Overall, the greatest relative loss in root weight was for ‘Excel’ and lowest for ‘Ntollo’. Effects on storage root weights were similar in the four- and six-bud treatments, with the greatest relative loss recorded for ‘Agric-rouge’ and no effect for ‘Oboul-doux’ or ‘TMS92/0326’. In the two-bud treatment, the greatest storage root weight was for ‘Adjatidaho’ and lowest for ‘TMS92/0326’, and the greatest relative loss in storage root weight was for ‘Excel’ and the lowest was for ‘Atinwewe’.

**Table 5:** Effect of viral dose (number of buds inoculated) on cassava storage root weight.

Cultivar	Storage root weight						
	Six buds		Four buds		Two buds		Control
	Mean ± SE	Loss %	Mean ± SE	Loss %	Mean ± SE	Loss %	Mean ± SE
Adjatidaho	267.00 ± 50.06	46.8	207.66 ± 41.12	58.63	200.00±82.03	60.15	493,33±31.60
92B/0057	111.33 ± 19.71	61.56	158.33 ±18.64	45.33	150.33±20.50	48.10	289.66±12.27
Agric-rouge	107.66 ±30.99	62.00	95.00 ±30.96	66.47	228.66±62.61	19.29	283.33 ±34.17
Atinwewe	181.33 ± 30.90	19.88	100.66 ±27.33	55.52	196.00±25.94	13.40	226.33 ± 16.71
BEN/86052	104.00 ±3.49	61.24	144.00 ± 11.33	46.33	191.33±10.56	28.69	268.33 ± 18.37
Oboul-doux	134.66 ± 50.43	9.41	153.00 ± 51.39	0	109.00±36.77	26.67	148.66 ± 17.52
Ntollo	214.33 ±45.36	0	123.66 ±18.84	31.04	102.33±16.56	42.93	179.33 ± 11.05
TME7	86.66 ± 6.22	66.75	111.00 ±3.29	57.41	119.33 ± 2.59	54.22	260.66 ±1.93
TMS92/0326	72.00 ± 32.93	44.61	170.66 ± 76.69	0	49.00 ± 17.72	62.30	130.00 ± 34.290
Excel	59.00 ± 4.42	71.26	75.33 ±2.79	63.31	66.00 ± 2.85	67.85	205.33 ± 34.29
**Overall**	**133.797**	**42.40**	**133.93**	**38.98**	**141.198**	**42.36**	**249.363**
**LSD**	**155.3662**	**–**	**166.6866**	**–**	**173.334**	**–**	**93.23**
**Khi**^**2**^ **(LR)**	**15.02**	**–**	**6.67**	**–**	**16.89**	**–**	**44.32**
***P***	**0.0001**	**–**	**0.0098**	**–**	**<0.0001**	**–**	**<0.0001**

## Discussion

Whereas levels of susceptibility to CMD in some cassava cultivars were previously quantified based on disease severity index and yield loss [20], our finding that disease severity among cultivars varied with plant age at inoculation indicates that this factor may be key in plant responses to CMD, although it is possible that these differences in disease severity may be related to environmental conditions. The response of an infected plant to young age is different from that infested in old age [21]. Thus, CMD tends to be active in infected plants at a young age. As the plant ages, it develops self-defense against the virus. Monde et al. [22] have also made similar observations on by screening cassava for resistance to cassava mosaic disease through grafting and whitefly inoculation. It is therefore important to know the susceptible age of cassava plants for a good inoculation protocols establishment. This makes glasshouse screening more accurate and precise than fields where plant age at infestation is an unknown factor [9].

We also found that disease development in cassava plants varied with inoculum dose, where an inoculum dose of two buds in 8-week-old plants was sufficient to induce expression of disease symptoms in ‘TMS92/0326’, ‘Excel’, and ‘Oboul-doux’. Some cultivars in our experiment developed symptoms with an inoculum dose of two buds, but others only developed symptoms with four or six buds, depending on plant age at inoculation. The inoculum dose is the second factor that influences the response of cassava cultivars. This factor has also been evaluated under glasshouse on other species such as potato [23], tomato [9], but which reveals to be determinants in their response. However, there is a strong interaction between the dose of the inoculum and the plant age at infestation, and this interaction was also reported by Difonzo et al. [23] on potato. The association of disease severity in ‘TMS92/0326’, ‘Oboul-doux’, and ‘Excel’ with treatments 8W2B, 8W6B, 10W2B, and 12W2B, and in ‘Ntollo’, ‘Adjatidaho’, ‘92B/0057’, and ‘BEN/86052’ with treatments 8W4B, 10W2B, 10W4B, 10W6B, 12W4B, and 12W6B indicates these cultivars were susceptible at all ages and inoculum doses used in our experiment. However, plant age at infestation and inoculation pressure can have major effects on the severity of the induced disease symptoms [10, 24]. Showing that effects of infection dose-response and age at inoculation varied among the cultivars, indicates that the susceptibility of the cultivar to an inoculum dose and plant age at infestation is related to genetic background (genotype) of the cultivar. Also, Kaweesi et al. [25] have evaluated cassava cultivars for cassava brown streak disease based on symptom expression and virus load and came to the conclusion that the response differed among cassava cultivars.

As the degree of cassava susceptibility to CMD varies with germplasm [26], our cultivar differences, in terms of disease symptom expression relative to time after inoculation and to inoculum dose, were probably a result of genotypic differences. Among the cultivars assessed in this current study, the first appearance of disease symptoms following inoculation ranged between 2 and 8 weeks. These differences in symptom development may also depend on environmental conditions (temperature, hygrometry), and similarly, disease severity may be influenced by the environment in which cassava is cultivated [27]. Disease symptoms were not observed in ‘TME7’, ‘Atinwewe’, or ‘Agric-rouge’ at any inoculum dose used in our study, confirming their resistance to CMD [16]. The cultivars ‘TME7’ and ‘Agric-rouge’ have already shown CMD2 type resistance in other studies [16, 28]. However, the inoculation methods could also influent disease symptoms appearance in CMD2-type resistance cultivars. It is the case of cultivar TME7, which showed moderate symptoms of CMD, inoculated by microparticle bombardment with infectious clones [13]. The absence of CMD symptoms in these resistant cultivars in our experiment would be related to environment effect as it has been demonstrated in other studies that the environment influences the expression of the gene [6, 29, 30]. Importantly, this study demonstrates that resistance in these cultivars is confirmed at inoculum doses greater than six buds.

We found that the inoculum dose affected the number of storage roots; in contrast to Elegba et al. [31] who found that CMD infection level did not affect the number of roots in a field environment. Additionally, we found that virus inoculation affected the number of roots in asymptomatic cultivars, possibly due to the timing of inoculation (2–3 months). A previous study showed differences in root weight between infected and uninfected plants, irrespective of inoculum pressure [32]; this agrees with our findings that viral infection reduced root weight. Lower root weight in inoculated plants may be explained by the decreased accumulation of starch in roots under disease pressure, possibly due to a reduction in photosynthesis [31]. This variation among cultivars in root weight and number with age at inoculation may be due to differences in the age at which they are susceptible to CMD. For example, Bisimwa et al. [33] showed CMD infection caused huge yield losses in susceptible cultivars during the first three months after planting, while Kovács et al. [34] and Gardner et al. [35] showed that plant age affects expression of other viral diseases in plants.

## Conclusion

Our results showed that resistance of cassava cultivars to CMD varies with inoculum dose and timing of infection; this will allow appropriate cultivars to be deployed in each production zone based on disease prevalence. Virus inoculation of susceptible cultivars negatively affected cassava root system demonstrating the likely impact on yields. Three cultivars (‘TMS92/0326’, ‘Oboul-doux’, and ‘Excel’) were susceptible at the two-bud inoculum dose, regardless of plant age at inoculation; however, the overall effect of plant age at inoculation was a key factor in the susceptibility of cassava cultivars to CMD.

## Supporting information

S1 File Phytosanitary certificate of plant material. Includes detailed on the tractability.
(PDF)

S2 File Gels support the results generated in Table 2. (PDF)

S1 Table Principal component analysis (PCA) of plant susceptibility with infection level and age at inoculation. Includes detailed and summary tables of PCA.
(DOCX)

S2 Table Analysis of variance (ANOVA) of cassava root numbers. Includes detailed and summary tables of ANOVA of treatment differences, with 95% confidence intervals.
(DOCX)

S3 Table Log-linear regression of cassava root weight. Includes a regression prediction plot and Type II analysis.
(DOCX)

S1 Raw images Original uncropped images underlying the gel results reported in the Fig 4. (PDF)

S2 Raw images Original uncropped images underlying the gel results reported in S2 File. (PDF)
