# Supplementary material for: Response of cassava cultivars to African cassava mosaic virus infection across a range of inoculum doses and plant ages
Source: PLoS One. 2019 Dec 23;14(12):e0226783. doi: 10.1371/journal.pone.0226783 (PMC6927654; doi:10.1371/journal.pone.0226783)
Supplement: S1 File — Includes detailed on the tractability. (PDF) [file pone.0226783.s001.pdf]

REPUBLIQUE DU BENIN<br>MINISTERE DE L'AGRICULTURE<br>DE L'ELEVAGE ET DE LA PECHE<br>**DIRECTION DE LA PRODUCTION VEGETALE**<br>1 **SERVICE DE LA PROTECTION DES VEGETAUX ET DU CONTROLE PHYTOSANITAIRE**<br>01 BP 58 Porto-Novo<br>Tél. : +229 20 21 32 90 / 20 21 32 93 - Fax : +229 20 21 44 13<br>spv.benin@yahoo.fr	2 **CERTIFICAT PHYTOSANITAIRE**<br>N° 0002975 /6/SPVCP/FCP/AE-B/<br>1 **Le Service de la Protection des Végétaux et du Contrôle Phytosanitaire du Bénin**<br>Au<br>Service de la Protection des Végétaux de<br>CAMEROUN<br>3 Lieu d'origine BENIN
4 **Nom et adresse de l'expéditeur**<br>M. HOUNGUE ANANI JEROME<br>C/ SB AGORI ZOGBADJE<br>TEL : +229 95610998.<br>BENIN	5 Nom et adresse du destinataire<br>M. HOUNGUE ANANI JEROME<br>UNIVERSITE DE YAOUNDE 1<br>TEL: +237 677702109<br>CAMEROUN
6 **Moyen de transport déclaré**<br>VOIE AERIENNE: ASKY DU 23/09/2016	7 Point d'entrée déclaré YAOUNDE
8 **Marques des colis: nombre et nature des colis** : nom commun du produit/organisme animal : nom scientifique **des plantes/organismes animaux**<br>**01** COLIS DE MATERIEL VEGETAL DE PROPAGATION::<br>- BOUTURES DE MANIOC: ***Manihot esculenta***	9 Quantité déclarée<br>**03,348 Kg**

10 **Il est certifié que les végétaux ou produits végétaux ou organismes animaux décrits ci-dessus**
- **ont été inspectés suivant des procédures adaptées, et**
- **estimés indemnes d'ennemis visés par la réglementation et pratiquement indemnes d'autres ennemis dangereux, et**
- **sont jugés conformes à la réglementation phytosanitaire en vigueur dans le pays importateur.**

REPUBLIQUE DU BENIN • POSTE DE CONTRÔLE PHYTOSANITAIRE DE L'AEROPORT 2 • MAEP • DPV • SPVCP

TIMBRE FISCAL 500 FRANCS G06766781 REPUBLIQUE DU BENIN

11 **Déclaration supplémentaire** NEANT

**TRAITEMENT DE DESINFECTATION ET/OU DE DESINFECTION**	18 Lieu de délivrance COTONOU
12 **Traitement** NEANT	19 Date Le 22 SEPTEMBRE 2016.
13 **Produit chimique (matière active)** NEANT | 14 Durée et température NEANT	20 Nom et signature du Fonctionnaire autorisé<br>B. HOUETONGNON
15 **Concentration** NEANT | 16 Date NEANT	21 Cachet de l'organisation<br>REPUBLIQUE DU BENIN • POSTE DE CONTRÔLE PHYTOSANITAIRE DE L'AEROPORT 2 • MAEP • DPV • SPVCP
17 Renseignements complémentaires NEANT	

NB : Le certificat phytosanitaire n'est délivré que sur la base d'un contrôle de la marchandise à exporter en tenant compte des exigences phytosanitaires du pays importateur. Il est subordonné à l'autorisation de l'importation attestée par le Permis d'importation du pays importateur

Scanned by CamScanner

1 Plant Protection Organization of
to Plant Protection Organization (s) of
Organization de Protection Fitosanitaria de
A Organizacion(es) de Protection Fitosanitaria de
Pflanzenschutzdienst(e) von
an Pflanzenschuftdienst (e) von

2 **PHYTOSANITARY CERTIFICATE**
CERTIFICADO FITOSANITARIO
PFLANZENGESUNDHEITSZEUGNIS

3 Place of origin
Lugar de origen
Ursprugsort

4 Name and address of exporter
Nombre y direccion del exportator
Name und Adresses des Absenders

5 Declared name and adress of congnee
Nombre y direccion declaradors del destinatario
Name und Adresse des angegebenen Emplangers

6 Declared mens of conveyance
Medios de transporte declarados
Angegebenes Transportmittel

7 Declared point of entry
Punto de entrada declarado
Angegebener Grenzübertrittsot

8 Distinguishing marks ; number and description or packges name of produce ; botanical name of plants
Marcas distintas ; numero y descripcon de los bultos, nombre del producto; nombre botanico de las plantas
Unlersheidungsmerkmate ; Zahl und Art der Packstucke; Name des Erzeugnisses; botanisher Name der Pilanzen

9 Quantity declared
Cantidad declarade
Angegebene Menge

10 This is to certify that the plants or plant products described above
- have been inspected according to appropriate procedures ; and
- are considered to be free from quaratine pests; ad patically free from other injurious pests ; and that they
- are considered to confrom with the current phytosanitary regulations of the importing country

Por la presente se certifica que las plantas o productors vegetales
- se han inspeccionado de acuerdo con los procedimientos adecuados, y
- se consideran exentos de plagas de curentena y praticamente exentos de otras plagas nocivas ; y que
- se considera que se ajustan a las disposiciones fitosanitas vigentes en el pais importador

Hiermit wrird bescheinigt, daв die oben beschnebenen pllanzen oder pllanzerzenerzeugnisse
- nach geeigneten Verfahren untersucht worden sind und
- frei von Quarantaneschadorganismen und praktisch rei on anderen getahrtichen Schadorganisrmen befunden wurden, und daв sie
- ais den besleheden Pllanzenschutzvorschriften des Bestimmungslandes enisprechend angesehen werden

11 Addtionel declaration
Declarcion suplementaia
Zusatzliche Erklarung

DESINFESTATION AND/OR DISINFECTION TRETMENT
TRATAMIENTO DE DESINFESTACION O DESIFECCION
ENTSEUCHUNG UND/ORDER DESINFIZIERUG

12 Treatment
Tratamiento
Behadlung

13 Chemical (active ingredient)
Preparado quimico (ingrediente acttivo)
Chemikalie (wirkstoff)

14 Duration and temperature
Duraction y temperatura
Dauer und Tempareratur

15 Concentration
Concentration
Konzentration

16 Date
Fecha
Datum

17 Additional information
Informacion adicional
Sostige Anzaben

18 Place of issue
Lugar de expedicion
Ort der Ausstellung

19 Date
Fecha
Datum

20 Name and signature of autorized officer
Nombre y firma del fun cioario autorizado
name und Unterschrift des amtlichen Beauftragten

21 Stamp of Organization
Selio de la Organizacion
Dienstsiegel

Scanned by CamScanner
