# Supplementary material for: Response of cassava cultivars to African cassava mosaic virus infection across a range of inoculum doses and plant ages
Source: PLoS One. 2019 Dec 23;14(12):e0226783. doi: 10.1371/journal.pone.0226783 (PMC6927654; doi:10.1371/journal.pone.0226783)

This is PCR analysis in some cultivars to ACMV as reported in the table 2. C+: control positive; W: week; B: buds; ACMV: African cassava mosaic virus; 8W2B: Plants inoculated at the age of eight weeks with two buds; 8W4B: Plants inoculated at the age of eight weeks with four buds; 8W6B: Plants inoculated at the age of eight weeks with six buds; 10W2B: Plants inoculated at the age of ten weeks with two buds; 10W4B: Plants inoculated at the age of ten weeks with four buds; 10W6B: Plants inoculated at the age of ten weeks with six buds; 12W2B: Plants inoculated at the age of twelve weeks with two buds; 12W4B: Plants inoculated at the age of twelve weeks with four buds; 12W6B: Plants inoculated at the age of twelve weeks with six buds.

Cultivars TMS/0326 and Agric-rouge

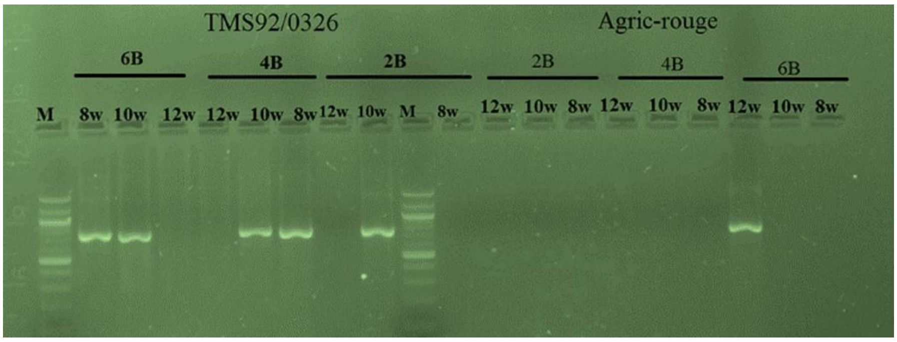

Cultivar TME7

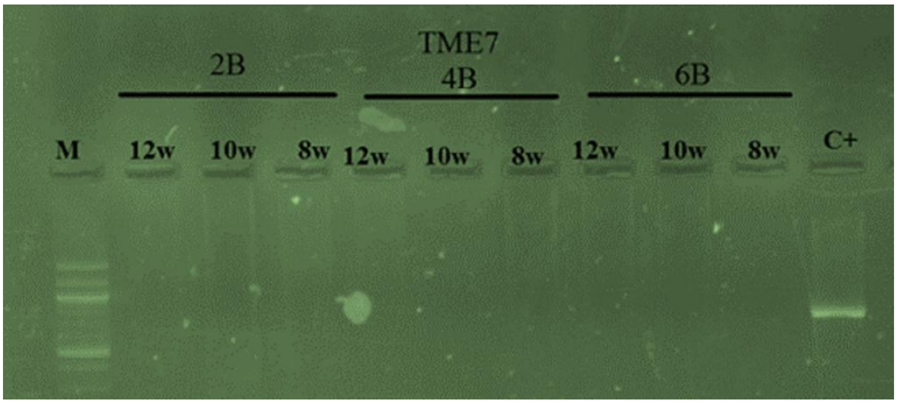

Cultivar Atinwewe

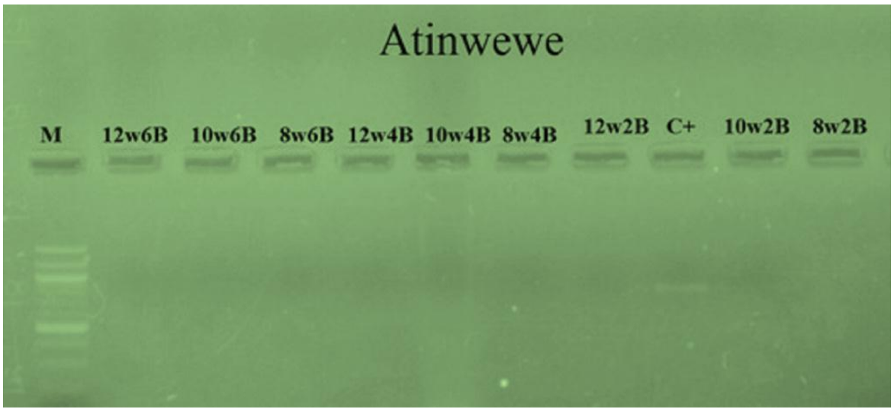

Cultivar Ntollo

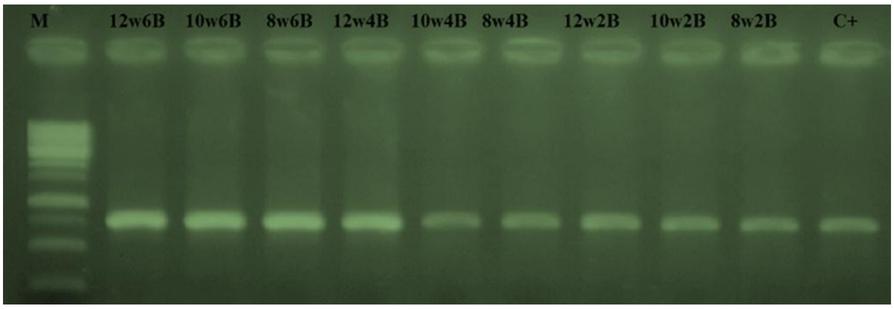

Supplement: S2 File — (PDF) [file pone.0226783.s002.pdf]
