# Supplementary material for: Response of cassava cultivars to African cassava mosaic virus infection across a range of inoculum doses and plant ages
Source: PLoS One. 2019 Dec 23;14(12):e0226783. doi: 10.1371/journal.pone.0226783 (PMC6927654; doi:10.1371/journal.pone.0226783)
Supplement: S1 Table — Includes detailed and summary tables of PCA. (DOCX) [file pone.0226783.s003.docx]

**Principal Component Analysis:**

**Eigenvalues**

|  | PC1 | PC 2 | PC 3 | PC 4 | PC5 | PC 6 | PC 7 |
| --- | --- | --- | --- | --- | --- | --- | --- |
| Eigenvalue | 5.8861 | 1.1213 | 1.0417 | 0.5791 | 0.2254 | 0.0892 | 0.0573 |
| Variability (%) | 65.4009 | 12.4594 | 11.5741 | 6.4343 | 2.5039 | 0.9909 | 0.6363 |
| Cumulative % | 65.4009 | 77.8604 | 89.4345 | 95.8688 | 98.3727 | 99.3637 | 100.0000 |

**Correlations between variables and factors.**

| Treatments | PC 1 | PC 2 | PC 3 |
| --- | --- | --- | --- |
| 8W2B | 0.5586 | 0.5872 | -0.5590 |
| 8W4B | 0.8216 | -0.0797 | -0.4564 |
| 8W6B | 0.9437 | 0.2952 | 0.0000 |
| 10W2B | 0.9521 | 0.0007 | 0.0000 |
| 10W4B | 0.8458 | -0.4441 | 0.0000 |
| 10W6B | 0.7681 | -0.2797 | 0.0000 |
| 12W2B | 0.5586 | 0.5872 | 0.5590 |
| 12W4B | 0.8216 | -0.0797 | 0.4564 |
| 12W6B | 0.8998 | -0.2378 | 0.0000 |

**Squared cosines of the variables**

|  | F1 | F2 | F3 |
| --- | --- | --- | --- |
| 8W2B | 0.3121 | **0.3448** | 0.3125 |
| 8W4B | **0.6750** | 0.0063 | 0.2083 |
| 8W6B | **0.8905** | 0.0872 | 0.0000 |
| 10W2B | **0.9064** | 0.0000 | 0.0000 |
| 10W4B | **0.7154** | 0.1972 | 0.0000 |
| 10W6B | **0.5899** | 0.0782 | 0.0000 |
| 12W2B | 0.3121 | **0.3448** | 0.3125 |
| 12W4B | **0.6750** | 0.0063 | 0.2083 |
| 12W6B | **0.8097** | 0.0565 | 0.0000 |

*Values in bold correspond for each variable to the factor for which the squared cosine is the largest*
