# Supplementary material for: Response of cassava cultivars to African cassava mosaic virus infection across a range of inoculum doses and plant ages
Source: PLoS One. 2019 Dec 23;14(12):e0226783. doi: 10.1371/journal.pone.0226783 (PMC6927654; doi:10.1371/journal.pone.0226783)
Supplement: S2 Table — Includes detailed and summary tables of ANOVA of treatment differences, with 95% confidence intervals. (DOCX) [file pone.0226783.s004.docx]

## **Effect of inoculum dose on storage roots number**

**Analysis of variance (By using 2 buds as inoculum dose):**

| Source | DF | Sum of squares | Mean squares | F | Pr > F |
| --- | --- | --- | --- | --- | --- |
| Model | 9 | 110.8000 | 12.3111 | 13.2846 | ˂0.0001 |
| Error | 20 | 40.6667 | 2.0333 |  |  |
| Corrected Total | 29 | 151.4667 |  |  |  |
| *Computed against model Y=Mean(Y)* | | |  |  |  |

**Cultivars / Fisher (LSD) / Analysis of the differences between cultivars based on storage root number with a confidence interval of 95%:**

| Category | LS means  (Root storage number) | Groups | | | | |
| --- | --- | --- | --- | --- | --- | --- |
| BEN/86052 | 7.3333 | A |  |  |  |  |
| Adjatindaho | 6.6667 | A | B |  |  |  |
| TMS92B/0057 | 6.3333 | A | B |  |  |  |
| Agric-rouge | 6.0000 | A | B |  |  |  |
| TME7 | 5.0000 | A | B | C |  |  |
| Atinwewe | 4.3333 |  | B | C | D |  |
| Ntollo | 3.0000 |  |  | C | D | E |
| Excel | 2.6667 |  |  | C | D | E |
| TMS92/0326 | 2.3333 |  |  |  | D | E |
| Oboul-doux | 1.6667 |  |  |  |  | E |

**Analysis of variance (By using 4 buds as inoculum dose)**

| Source | DF | Sum of squares | Mean squares | F | Pr > F |
| --- | --- | --- | --- | --- | --- |
| Model | 9 | 44.5333 | 4.9481 | 4.7278 | 0.0001 |
| Error | 20 | 51.3333 | 2.5667 |  |  |
| Corrected Total | 29 | 95.8667 |  |  |  |
| *Computed against model Y=Mean(Y)* | | |  |  |  |

**Cultivars / Fisher (LSD) / Analysis of the differences between cultivars based on storage root number with a confidence interval of 95%**

| Category | LS means  (Root storage number) | Groups | |
| --- | --- | --- | --- |
| Adjatindaho | 5.6667 | A |  |
| Agric-rouge | 5.6667 | A |  |
| TME7 | 5.0000 | A | B |
| TMS92B/0057 | 5.0000 | A | B |
| BEN/86052 | 4.6667 | A | B |
| Atinwewe | 3.6667 | A | B |
| Excel | 3.3333 | A | B |
| TMS92/0326 | 2.6667 |  | B |
| Ntollo | 2.6667 |  | B |
| Oboul-doux | 2.3333 |  | B |

**Analysis of variance (By using 6 buds as inoculum dose)**

| Source | DF | Sum of squares | Mean squares | F | Pr > F |
| --- | --- | --- | --- | --- | --- |
| Model | 9 | 28.1333 | 3.1259 | 3.9778 | 0.0007 |
| Error | 20 | 25.3333 | 1.2667 |  |  |
| Corrected Total | 29 | 53.4667 |  |  |  |
| *Computed against model Y=Mean(Y)* | | |  |  |  |

**Cultivars / Fisher (LSD) / Analysis of the differences between cultivars based on storage root number with a confidence interval of 95%**

| Category | LS means  (Root storage number) | Groups | | | | |
| --- | --- | --- | --- | --- | --- | --- |
| BEN/86052 | 4.3333 | A |  |  |  |  |
| Agric-rouge | 4.0000 | A | B |  |  |  |
| TME7 | 3.6667 | A | B | C |  |  |
| Adjatindaho | 3.3333 | A | B | C | D |  |
| TMS92B/0057 | 3.3333 | A | B | C | D |  |
| Excel | 2.6667 | A | B | C | D | E |
| Atinwewe | 2.3333 |  | B | C | D | E |
| TMS92/0326 | 2.0000 |  |  | C | D | E |
| Oboul-doux | 1.6667 |  |  |  | D | E |
| Ntollo | 1.3333 |  |  |  |  | E |

**Analysis of variance (In the control plants)**

| Source | DF | Sum of squares | Mean squares | F | Pr > F |
| --- | --- | --- | --- | --- | --- |
| Model | 9 | 40.8333 | 4.5370 | 3.0322 | 0.0057 |
| Error | 20 | 33.3333 | 1.6667 |  |  |
| Corrected Total | 29 | 74.1667 |  |  |  |
| *Computed against model Y=Mean(Y)* | | |  |  |  |

**Cultivars / Fisher (LSD) / Analysis of the differences between cultivars based on storage root number with a confidence interval of 95%**

| Category | LS means  (Root storage number) | Groups | | | |
| --- | --- | --- | --- | --- | --- |
| TMS92B/0057 | 9.6667 | A |  |  |  |
| Agric-rouge | 9.3333 | A |  |  |  |
| BEN/86052 | 8.6667 | A | B |  |  |
| Excel | 8.3333 | A | B | C |  |
| Ntollo | 8.0000 | A | B | C | D |
| TME7 | 8.0000 | A | B | C | D |
| Adjatindaho | 7.0000 |  | B | C | D |
| Atinwewe | 7.0000 |  | B | C | D |
| TMS92/0326 | 6.3333 |  |  | C | D |
| Oboul-doux | 6.0000 |  |  |  | D |
