# Supplementary material for: Response of cassava cultivars to African cassava mosaic virus infection across a range of inoculum doses and plant ages
Source: PLoS One. 2019 Dec 23;14(12):e0226783. doi: 10.1371/journal.pone.0226783 (PMC6927654; doi:10.1371/journal.pone.0226783)
Supplement: S3 Table — Includes a regression prediction plot and Type II analysis. (DOCX) [file pone.0226783.s005.docx]

## **Effect of inoculum dose on storage root weight**

**Test of the null hypothesis H0: Y=Constant (By using 2 buds as inoculum dose)**

| Statistic | DF | Chi-square | Pr > Chi² |
| --- | --- | --- | --- |
| -2 Log(Likelihood) | 9 | 16.896581 | 0.0504 |
| Score | 9 | 3.74533259 | 0.9274 |
| Wald | 9 | 24.7411446 | 0.0033 |

**Type II analysis on storage root weight (By using 2 buds as inoculum dose)**

| Source | DF | Chi-square (LR) | Pr > LR |
| --- | --- | --- | --- |
| Cultivar | 9 | 16.8966 | < 0.0001 |

**Test of the null hypothesis H0: Y=Constant (By using 4 buds as inoculum dose)**

| Statistic | DF | Chi-square | Pr > Chi² |
| --- | --- | --- | --- |
| -2 Log(Likelihood) | 9 | 6.67875897 | 0.6705 |
| Score | 9 | 9.86868605 | 0.3612 |
| Wald | 9 | 7.57263554 | 0.5777 |

**Type II analysis on storage root weight (By using 4 buds as inoculum dose)**

| Source | DF | Chi-square (LR) | Pr > LR |
| --- | --- | --- | --- |
| Cultivar | 9 | 6.6788 | 0.0098 |

**Test of the null hypothesis H0: Y=Constant (By using 6 buds as inoculum dose)**

| Statistic | DF | Chi-square | Pr > Chi² |
| --- | --- | --- | --- |
| -2 Log(Likelihood) | 9 | 15.0201451 | 0.0904 |
| Score | 9 | -10.1815962 |  |
| Wald | 9 | 18.5685574 | 0.0291 |

**Type II analysis on storage root weight (By using 6 buds as inoculum dose)**

| Source | DF | Chi-square (LR) | Pr > LR |
| --- | --- | --- | --- |
| Cultivar | 9 | 15.0201 | 0.0001 |

**Test of the null hypothesis H0: Y=Constant (With Control plants)**

| Statistic | DF | Chi-square | Pr > Chi² |
| --- | --- | --- | --- |
| -2 Log(Likelihood) | 9 | 44.3298161 | < 0.0001 |
| Score | 9 | 70.5184629 | < 0.0001 |
| Wald | 9 | 95.8920979 | < 0.0001 |

**Type II analysis** **on storage root weight** (**with control plants**)

| Source | DF | Chi-square (LR) | Pr > LR |
| --- | --- | --- | --- |
| Cultivar | 9 | 44.3298 | < 0.0001 |
