# Supplementary material for: Response of cassava cultivars to African cassava mosaic virus infection across a range of inoculum doses and plant ages
Source: PLoS One. 2019 Dec 23;14(12):e0226783. doi: 10.1371/journal.pone.0226783 (PMC6927654; doi:10.1371/journal.pone.0226783)
Supplement: S1 Raw images — (PDF) [file pone.0226783.s006.pdf]

ACMV

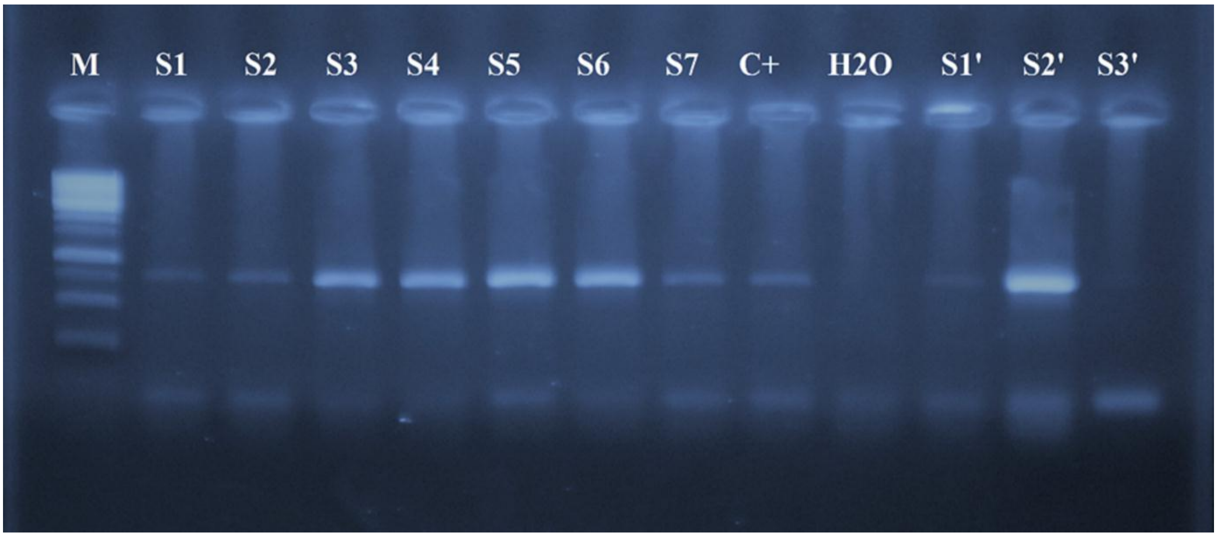

From this original images was generated the Fig 4 a in the main manuscript. The S1', S2', and S3' were the replicate of the sample S1, S2, S3 respectively.

EACMV

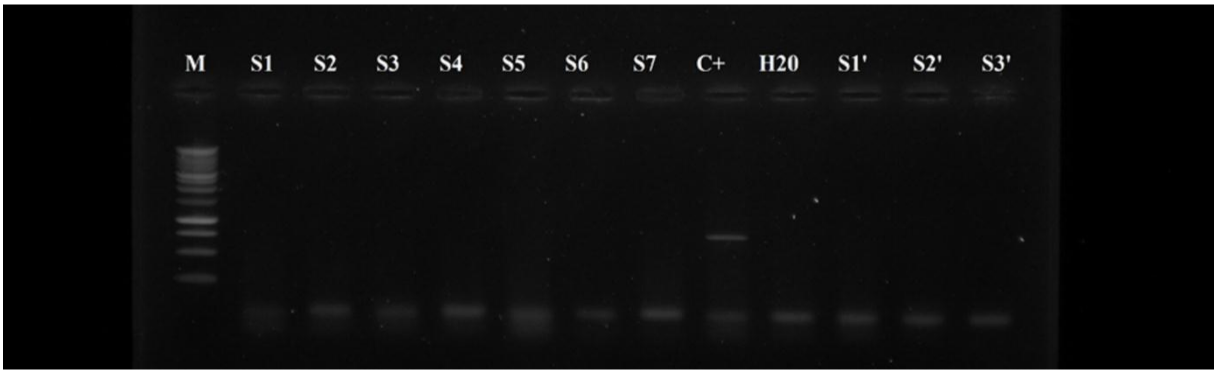

From this original image was generated the Fig 4b in the main manuscript. The S1', S2', and S3' were the replicate of the sample S1, S2, S3 respectively.
