# Supplementary material for: Response of cassava cultivars to African cassava mosaic virus infection across a range of inoculum doses and plant ages
Source: PLoS One. 2019 Dec 23;14(12):e0226783. doi: 10.1371/journal.pone.0226783 (PMC6927654; doi:10.1371/journal.pone.0226783)

These are the original uncropped and unjusted gels suppoting the S2_File. C+: Control positive; W: week; B: buds; ACMV: African cassava mosaic virus; 8W2B: Plants inoculated at the age of eight weeks with two buds; 8W4B: Plants inoculated at the age of eight weeks with four buds; 8W6B: Plants inoculated at the age of eight weeks with six buds; 10W2B: Plants inoculated at the age of ten weeks with two buds; 10W4B: Plants inoculated at the age of ten weeks with four buds; 10W6B: Plants inoculated at the age of ten weeks with six buds; 12W2B: Plants inoculated at the age of twelve weeks with two buds; 12W4B: Plants inoculated at the age of twelve weeks with four buds; 12W6B: Plants inoculated at the age of twelve weeks with six buds. X: Lanes not included.

Cultivar Atinwewe

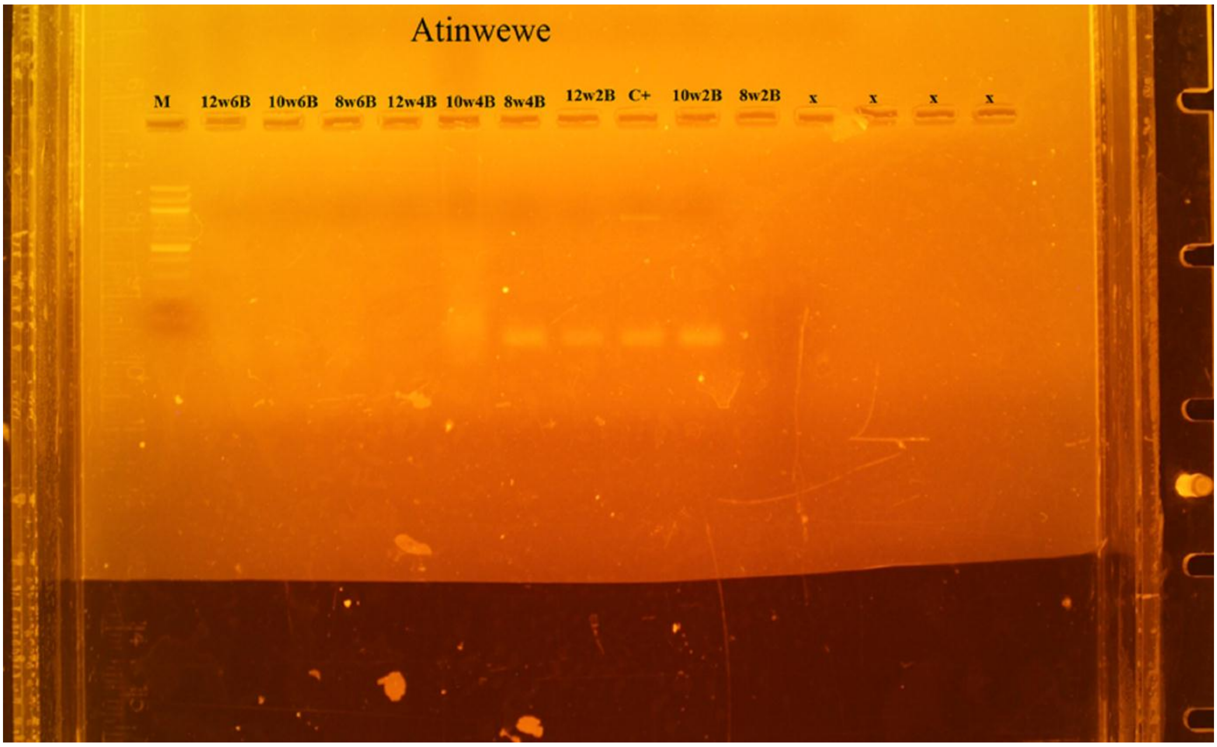

Cultivar TMS92/0326 and Agric-rouge

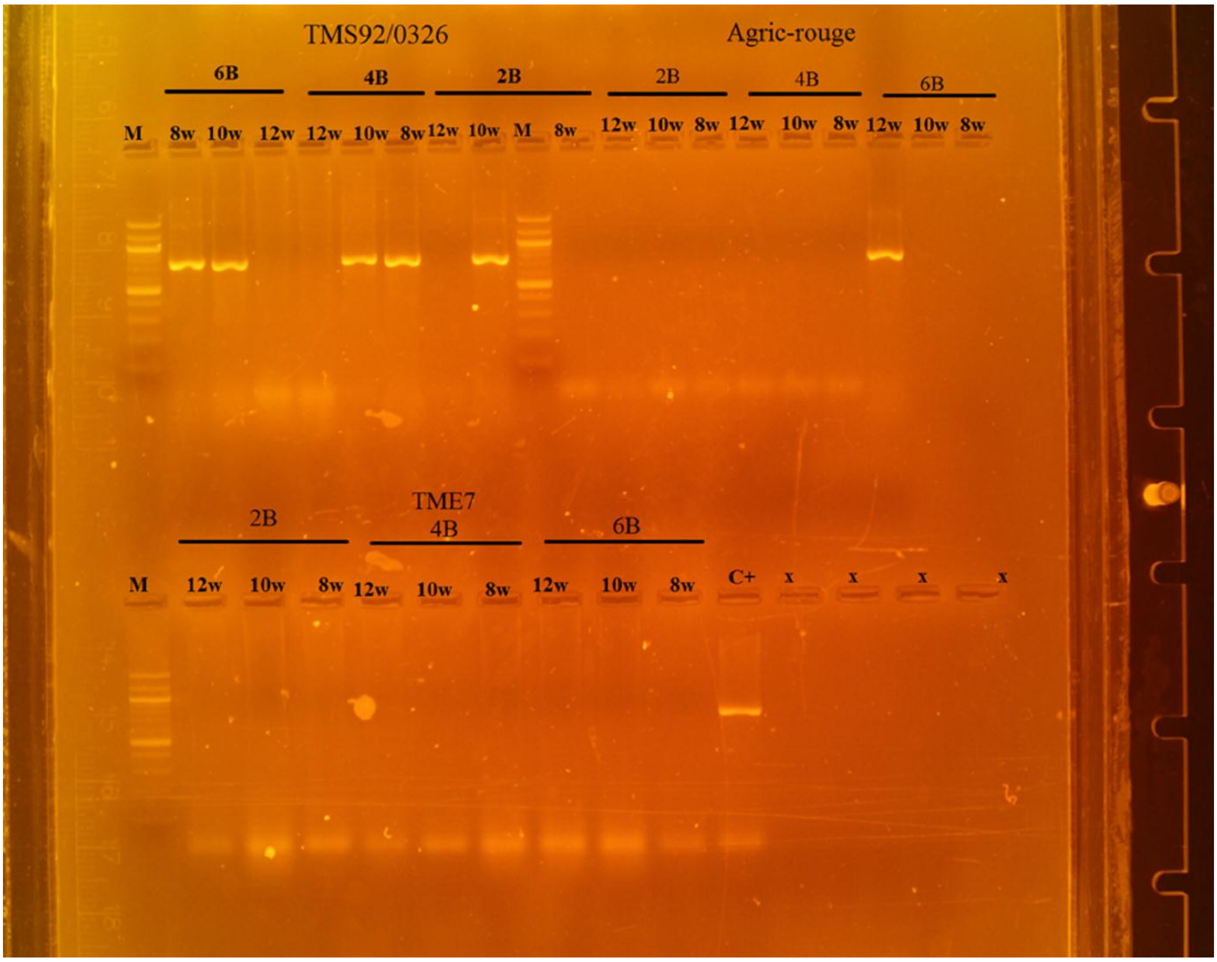

Cultivar Ntollo

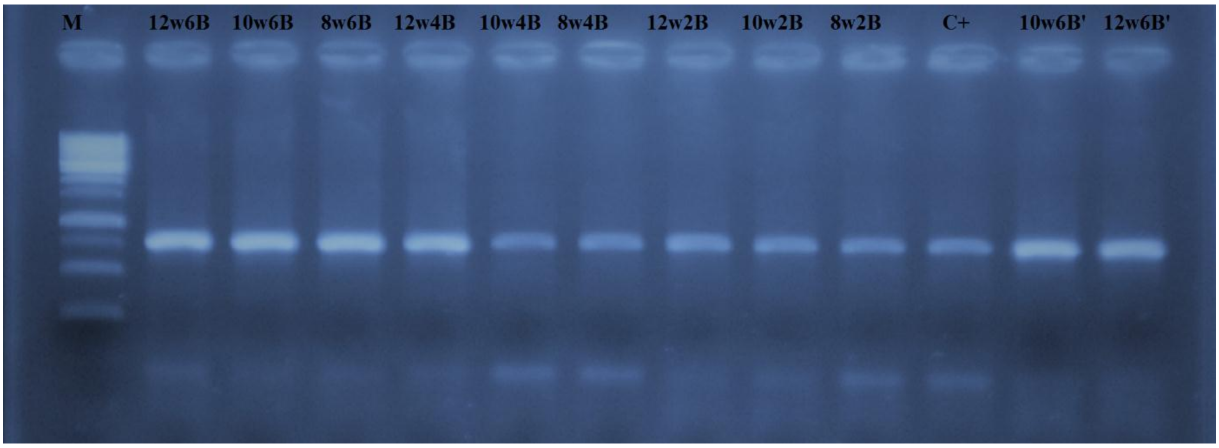

Supplement: S2 Raw images — (PDF) [file pone.0226783.s007.pdf]
